# The Heartrate Reaction to Acute Stress in Horned Passalus Beetles (*Odontotaenius disjunctus*) is Negatively Affected by a Naturally-Occurring Nematode Parasite

**DOI:** 10.3390/insects8040110

**Published:** 2017-10-18

**Authors:** Andrew K. Davis, Brandon Coogler, Isaac Johnson

**Affiliations:** Odum School of Ecology, University of Georgia, Athens, GA 30602, USA; bmcoogler@uga.edu (B.C.); isaac.johnson25@uga.edu (I.J.)

**Keywords:** insect stress, heartrate, passalus beetle, *Odontotaenius disjunctus*, nematodes, *Chondronema passali*

## Abstract

There are many events in the lives of insects where rapid, effective stress reactions are needed, including fighting conspecifics to defend territories, evading predators, and responding to wounds. A key element of the stress reaction is elevation of heartrate (HR), for enhancing distribution of blood (hemolymph) to body compartments. We conducted two experiments designed to improve understanding of the insect stress reaction and how it is influenced by parasitism in a common beetle species (*Odontotaenius disjunctus*). By non-destructively observing heartbeat frequency before, during and after applying a stressor (physical restraint) for 10 min, we sought to determine: (1) the exact timing of the cardiac stress reaction; (2) the magnitude of heartrate elevation during stress; and (3) if the physiological response is affected by a naturally-occurring nematode parasite, *Chondronema passali*. Restraint caused a dramatic increase in heartrate, though not immediately; maximum HR was reached after approximately 8 min. Average heartrate went from 65.5 beats/min to a maximum of 81.5 (24.5% increase) in adults raised in the lab (*n* = 19). Using wild-caught adults (*n* = 77), average heartrates went from 54.9 beats/min to 74.2 (35.5% increase). When restraint was removed, HR declined after ~5 min, and reached baseline 50 min later. The nematode parasite did not affect baseline heartrates in either experiment, but in one, it retarded the heartrate elevation during stress, and in the other, it reduced the overall magnitude of the elevation. While we acknowledge that our results are based on comparisons of beetles with naturally-occurring parasite infections, these results indicate this parasite causes a modest reduction in host cardiac output during acute stress conditions.

## 1. Introduction

Insects, like all animals, are often faced with stressful events in their lives, such as evading predators, fighting with conspecifics and coping with injuries. All of these events require a functional stress response, which in vertebrates as well as invertebrates, is designed to elicit specific changes in physiological functions that temporarily promote survival during the stress event [1,2,3]. Such changes include heightened immunity [4,5,6], enhanced predator evasion [7] and importantly, increased cardiac output, which serves to increase blood flow (hemolymph in insects) and nutrients to muscles. Cardiac output can be gauged by monitoring heartbeat frequency, or the pulsations of the dorsal vessel (the insect ‘heart’), which distributes hemolymph throughout the insect. It has long been known that when insects are faced with a physically demanding activity, such as flight, the heart beats faster. In fact, one of the earliest examples of this comes from a study conducted in the 19th century; Newport [8] scraped the upper abdominal scales off of a hawk moth (*Sphinx ligustri*) to view the pulsations of the dorsal vessel. He noted when the specimen was at rest, the heart rate was 42–49 beats/min, and after the moth had “flown around his sitting-room” for several minutes it was 127–139 beats/min. Thus, insect heartrates become elevated during situations when greater blood distribution is needed.

Similar to the response to physical activity, a positive relationship between acute stress states and heartrate elevations in insects is evident in the entomological literature, although mostly indirectly. A considerable body of work has been completed showing how stress leads to increases in circulating levels of octopamine, the insect equivalent to adrenaline [9,10,11,12]. Meanwhile, other work has shown that heightened levels of octopamine has a stimulatory effect on insect heart contractions [13,14,15]. From these two separate bodies of work, one can logically surmise that acute stress (which increases octopamine) leads to an increase in heartrate in insects, though there are many questions about this relationship that could be examined further, and in a more direct manner. Specifically, investigating how heartrates of (intact) insects change while insects are subjected to a stress event would be useful. This would help to address a number of questions relating to the timing of the stress reaction. For example, how quickly does heartrate increase when stress begins, and how long does the stress effect last following the stress event? Also, how is this reaction affected by the health of the insect? Can insects harboring parasites react as well to stress?

**Figure 1:**
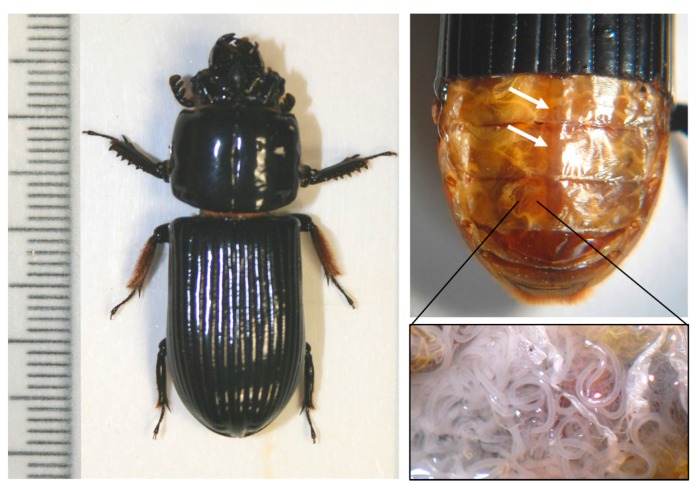
Intact horned passalus beetle, *Odontotaenius disjunctus*, left. Right top image shows a beetle with the lower portion of the elytra removed to allow visualization of the dorsal vessel (white arrows). A video of the dorsal vessel pumping is available in the Supplemental files. Lower right image shows nematode parasite, *Chondronema passali*, that inhabits the abdominal cavity of *O. disjunctus*.

We addressed the questions above using a natural host–parasite insect system that is ideal for investigations of insect stress and parasite effects. The horned passalus beetle, *Odontotaenius disjunctus* (Coleoptera: Passalidae, Illiger; Figure 1), is a medium-sized saproxylic insect that inhabits rotting hardwood logs in forests throughout the eastern seaboard of the United States [16,17,18]. It creates cavities within the logs, where it raises its larvae in the summer months. Since it consumes the wood as food, this species can be readily maintained in a laboratory setting by providing rotting wood. Importantly, it is host to a number of external and internal parasites [19], with one notable one being a nematode, *Chondronema passali* (Leidy), that lives in its abdominal cavity (Figure 1) [20]. This parasite is not well-studied, thus it is not known how it is transmitted (though probably from parent to offspring), and its taxonomy is not even clear.

This nematode is known to be highly prevalent in field collections of passalus beetles, with most collections having between 60 and 100% parasitism rates [16]. Moreover, it can be extremely abundant within its host, with beetles often harboring many hundreds, and sometimes thousands of worms. Despite this, the nematode seems to convey little outward negative effect to the host. Recent projects in our lab have begun to examine how this parasite affects its host [21,22,23], with particular attention given to behaviors and events in the lives of beetles where its impact might be felt most strongly, that is, during times of stress, when energy demand is high.

Here we conducted two experiments using field-collected passalus beetles, in which we monitored heartrates of intact specimens before, during and following a stressor in the laboratory (restraint). Moreover, we employed a non-destructive observational approach to directly count pulsations of the dorsal vessel of the living insect under a low-power microscope (see methods), even as the stress was applied, allowing us to know the exact timing of the heartrate elevations. This low-tech approach differs from that used by most previous investigators studying insect cardiac activity, where experiments often involve various electronic devices and probes attached to, or inserted into, the insect [24,25,26]. One prior study we are aware of has employed a similar, observational approach to monitor heartrate of a hemipteran, *Rhodnius prolixus* [27]. The goals of the current project were to: (1) document the magnitude and time course of the heartrate response during acute stress; and (2) determine if the *C. passali* nematodes influence either the resting heartrate, or the beetle heartrate during acute stress. The results from this project should be useful for improving understanding of the insect physiological stress response.

## 2. Methods

### 2.1. Beetle Collections

There were two collections of passalus beetles used for this project. For both collections the beetles were hand-collected from hardwood forests near the University of Georgia campus, in Athens, GA, USA. Beetles were extracted from rotting hardwood logs on the forest floor (using hatchets) and placed in plastic containers for transport back to the lab. There, they were housed individually in 900 mL plastic containers filled with moist, rotting hardwood. Beetles for the first experiment were a set of 19 specimens that were collected as larvae in August 2016, and housed in the lab until they became adult beetles (late October). We have found that larvae can be effectively reared using pulverized, moistened wood pulp [28]. When they became adults we replaced the wood pulp with actual pieces of hardwood. The second experiment used a collection of 80 adult beetles that were collected on 3 February 2017, and housed, as indicated above, in the lab until used for the project.

### 2.2. Measuring Heartrate

Each experiment involved measuring heartrate in individual beetles, which has been described previously [22]. Briefly, we removed a section of the elytra near the posterior end of the beetle (Figure 1) exactly one week prior to the experiment. The beetles were replaced into their housing containers following the dissecting, and were not disturbed during the week prior to the experiment. Removing the elytra allowed us to visualize the dorsal vessel under the translucent skin using a stereo-microscope (during the stress procedure, below) (Leica, Buffalo Grove, IL, USA). The pulsations of the vessel (i.e., the heart) can thus be readily counted (a video is provided in the Supplemental files). For recording heartrates before, during, and after the stress procedure, we counted heart beats over a 1 min period, and this rate (beats per minute) was used in all analyses (see below).

### 2.3. Stress Procedure

Both experiments involved applying a short-term (acute) stressor to individual beetles, which was the same in each experiment. Immediately before the stress was applied, individual beetles were removed from their housing container, and very quickly (i.e., less than 10 s) placed under the stereo-microscope to begin recording heartrate. This timing was important as we wanted to minimize any effect of handling on the first measurement, which we considered the ‘baseline’, or ‘resting’ heartrate. For this initial heartrate measure, the beetle was only gently grasped between the observer’s thumb and forefinger to keep it positioned under the microscope. While we recognize that even this handling could be enough to instigate a stress response, our thinking was that as long as we acted fast to begin the counting, the first measurement would still represent a modest ‘baseline’ rate, since it was initiated within seconds of removal from the housing. Preliminary work in our lab using an electronic heartrate measuring device [29] on unrestrained and restrained beetles has indicated that the heartrate response to handling does not begin increasing in earnest until 1–2 min after the beetle has been picked up [30]. Moreover, in the current experiment we began the actual stress procedure immediately after the first measurement, so that either way, we intended for the beetle to become stressed soon after this first measurement.

**Figure 2:**
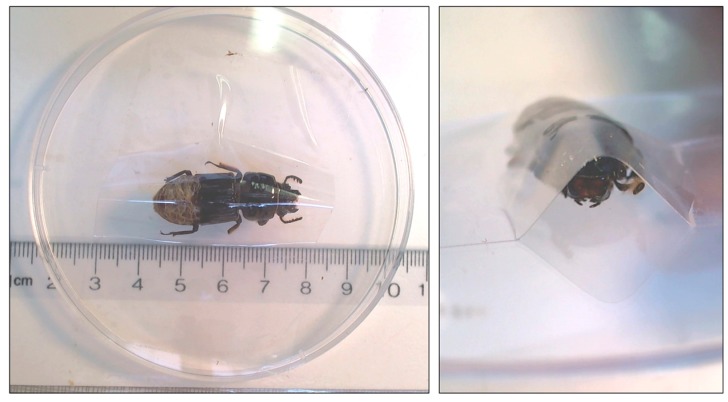
Photos of procedure used to apply restraint stress to beetles in both experiments. Each beetle was taped down to a petri dish lid using clear packing tape, which physically immobilized the body, head and legs of the specimen.

For the stress procedure, the beetle was placed right side-up on a petri dish cover and was then covered with clear packing tape so that the beetle was physically restrained (Figure 2). The petri cover and beetle were then returned to the stereo-microscope and we continued to record heartrates (the heart could still be seen through the clear tape). This allowed us to visualize, in real-time, the effect of physical restraint on heartrate. A video of the dorsal vessel pumping is provided in the Supplemental files. The restraint effect of the tape was such that the beetles could not move side-to-side, could not move their legs, and were pressed down onto the petri lid, so that this restraint was much more pronounced than the gentle handling used to position the beetle during the unrestrained measurements. Similar restraint procedures have been employed for stressing insects by other investigators [31]. Moreover, restraint is routinely used to impart stress in mammals within the biomedical and animal science fields e.g., [32,33,34]. We maintained the restraint stress for 10 min, while collecting heartrate data every 2 min.

### 2.4. Experiment Goals

The goals of our two experiments differed slightly. The first experiment was intended to elucidate the magnitude (i.e., of heartrate elevation) and the timecourse of the acute stress reaction, and to determine if this is affected by parasitism. Therefore in that project we collected heartrate data at baseline, during acute stress application, plus for 70 min after the tape was removed from the beetles (one measurement every 2 min) to determine the time to recover to baseline. For the recovery phase, the beetle was gently placed under the microscope during the heartrate measurement, then placed in an empty plastic container in the alternate minutes.

Based partly on the outcome of the first experiment (using 19 beetles), which showed the stress reaction is mainly influenced by parasites during the 10-min stress application itself (see results), the goal of the second experiment was to provide more data (i.e., more beetles, *n* = 77) for examining how parasites influence the *magnitude* of this immediate reaction. Thus, we collected heartrate before and during the stress procedure only in this experiment.

### 2.5. Parasite Assessment

At the completion of the experiments we weighed each beetle, then dissected it to determine sex (by the presence or absence of the male eadegus) and nematode parasitism status. The nematode, *C. passali*, lives in the abdominal cavity and can be readily seen when the cavity is opened and under a stereo-microscope (Figure 1). In the first experiment, there were 7 parasitized and 12 non-parasitized beetles. In the second, there were 63 parasitized and 14 non-parasitized beetles. The majority of our statistical analyses regarding the parasite focused only on the presence or absence of this nematode (i.e., parasitized or non-parasitized). However, we did consider how parasite load influenced baseline and stressed heartrates of beetles in our second experiment, where our sample size was larger. Parasite load was visually evaluated on a coarse scale of 0–3 for each beetle, where 0 equals no worms, 1 equals 1–10 worms, 2 equals 11–100 worms, and 3 equals more than 100 worms [21].

### 2.6. Data Analyses

Heartrate data for both the first and second experiment were normally-distributed, based on inspection of histograms. Given that two experiments were conducted, we first compared the heartrate metrics of beetles across both experiments. We specifically compared initial heartrate, maximum heartrate during stress, and the relative heartrate elevation (maximum heartrate as a percentage of the initial) between the first and second experiment. Since the variance between experiments was not equal for each metric (Levene’s test, *p* < 0.01 for all three), we used Mann-Whitney U tests for the comparisons.

Next, since the primary goal of the first project was to visualize the *timecourse* of the stress reaction, we calculated mean heartrates for each measurement (at baseline, stress and recovery) for graphical display. Then, we separately compared heartrates of beetles with and without *C. passali* parasites at baseline, during acute stress and during the recovery phases. We used ANOVA for the baseline measurement and repeated–measures ANOVA for the stress phase (*n* = 5 measurements) and the recovery phase (*n* = 35 measurements). In all models, we included beetle mass as a covariate. Beetle sex was initially included but was removed when it was found to be non-significant, and the models were re-run without sex. Importantly, nematode parasitism (parasitized, not parasitized) was a predictor in all three models.

For the second experiment we were primarily interested in the *magnitude* of the heartrate elevation during stress. Thus, for each beetle we determined the maximum HR during the stress application (i.e., maximum reading from all 5 stress readings), then converted this to a percentage of the initial HR. Then, we examined how maximum HR is affected by nematode parasitism (parasitized, not parasitized) using ANOVA. Beetle mass was included in each model.

Finally, we used beetles in this second collection to evaluate how *varying levels of parasitism* affected baseline and stressed heartrates. Here we used ANOVA to compare mean baseline and relative maximum heartrates across the 4 parasitism groups (see 2.5 Parasite Assessment, above). All analyses were conducted using the Statistica 12.1 software package (StatSoft, Tulsa, OK, USA).

## 3. Results

### 3.1. Comparisons between Experiments

Subjecting beetles to restraint caused a dramatic increase in heartrate in both experiments, although the magnitude of the increase varied significantly between experiments. The average initial heartrate also varied significantly between experiments (Table 1). Initial heartrate in experiment 1 went from 65.5 beats/min on average to 81.5, or an elevation of 24.5% (Table 1). In experiment 2, beetle heartrates went from an average of 54.9 beats/min to 74.2, or an elevation of 35.5%. All heartrate metrics (baseline, maximum and relative maximum) differed significantly between experiments based on Mann-Whitney U tests (*p* < 0.01 for all; Table 1).

### 3.2. Time Course of Stress Response

**Figure 3:**
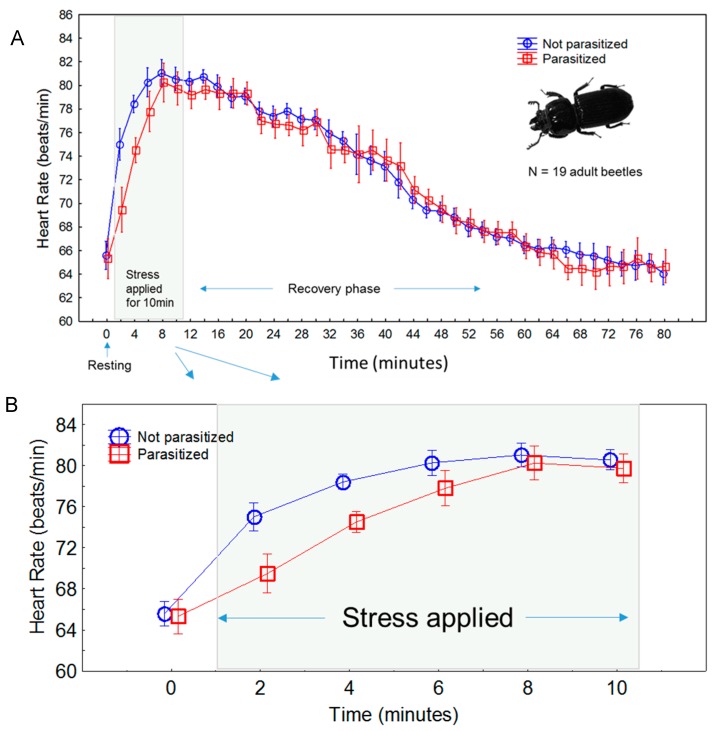
Results from experiment 1: (**A**) Time course of the acute stress response in 19 captive *O. disjunctus* specimens, based on heartrate readings, and the effects of the nematode parasite, *C. passali*, on the HR response. A resting, or baseline HR was obtained immediately after the beetle was removed from its housing. The restraint stress procedure followed (see Figure 2) for 10 min. Heartrate was recorded for 70 min after restraint ended to monitor recovery. Lower panel (**B**) shows an expanded view of the stress phase, where the impact of the parasite was most pronounced.

Plotting the mean HR across all measurements in experiment 1 provides a graphical representation of the complete acute stress reaction, including during the stress event, as well as during recovery (Figure 3). During the acute stress application, HR began rising at the first measurement, which was made at the start of the restraint (though the rate of elevation differs between parasitized and non-parasitized beetles, see below). The maximum HR was reached at the 4th measurement during stress, or after approximately 8 min of restraint. Based on visual inspection of Figure 3, we noted that when restraint was removed, the HR began to decline after ~5 min, and continued to gradually decline thereafter, reaching the baseline HR at close to the 60 min mark, or about 50 min after stress was removed.

### 3.3. Effects of Parasitism

For experiment 1 we separately evaluated the effects of nematode parasitism during the initial, stress, and recovery periods. After accounting for beetle mass, there was no difference in initial HR between parasitized and non-parasitized beetles (F_1,15_ = 0.08, *p* = 0.7776; Table 2). However, there was a significant effect of nematodes on the heartrate during stress (F_1,15_ = 22.64, *p* = 0.0003), and this effect was negative, as viewed from Figure 3. During the stress application, heartrates of parasitized beetles responded much slower to the stress, although they did appear to reach a similar maximum HR as that of the non-parasitized beetles. The significant Time*Parasitism interaction in this model also indicates that the two groups differed in trajectory, not necessarily in final magnitude. Finally, there was no effect of parasitism on the HR recovery phase (F_1,15_ = 0.38, *p* = 0.5476; Table 2). Collectively, these results indicate that beetles with the *C. passali* parasite are capable of mounting a physiological stress reaction (as measured by cardiac output), but a key difference is their cardiac elevation in the first 2–4 min of stress appears muted (Figure 3B) compared to non-parasitized individuals.

The effect of the nematode parasite in the second experiment, conducted using 77 wild-caught beetles, was less pronounced, but paralleled those of the first experiment. Baseline heartrates of beetles with and without the parasite were slightly different (Table 3) but when examined with ANOVA (with mass and parasitism as predictors) there was no effect of either mass or parasitism (*p* > 0.1 for both; Table 4). Meanwhile the model that examined relative HR elevation (maximum HR relative to baseline) showed clear effects of parasitism. When beetle sex and mass were accounted for, there was a significant (negative) effect of parasitism on HR elevation (F_1,74_ = 6.93, *p* = 0.0103; Table 4). The average stress-induced heartrate elevation in non-parasitized beetles was 44.6%, while in parasitized beetles it was 33.5% (Table 3). From visually gauging Table 3, it is clear that the relative elevation in heartrate was lower in parasitized beetles because their baseline heartrate was slightly higher in this collection, since maximum heartrate (beats/min) was similar between parasitism groups. We interpret these results to indicate that the nematode parasite does not influence the heartrate of the host during rest or regular day-to-day activity, but it appears to slightly reduce the cardiac output during a stressful situation.

**Figure 4:**
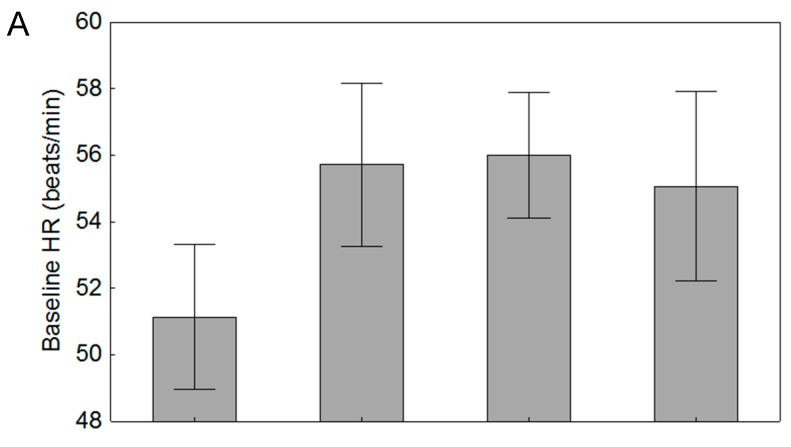

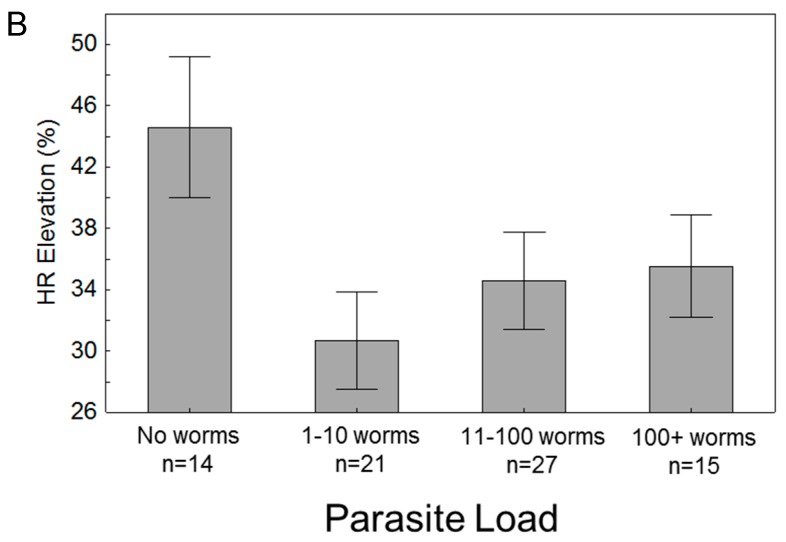
Comparison of (**A**) baseline and (**B**) stressed heartrates of beetles across parasite intensity groupings (see 2.5 Parasite Assessment) in the second experiment (*n* = 77 wild beetles). Whiskers represent standard errors of the means.

Evaluating how the nematode parasite load influenced heartrates of beetles did not change the conclusions above. There was no statistically significant variation detected in baseline heartrate across parasite intensity groupings (F_3,73_ = 0.78, *p* = 0.507; Figure 4). Meanwhile there was variation in stressed heartrates that approached significance (F_3,73_ = 2.34, *p* = 0.081; Figure 4). In both graphs, it appears that the varying levels of parasitism have similar effects on heartrates.

## 4. Discussion

The current project adds to a small but growing body of work (though mostly in vertebrates) that shows how parasites can influence their host’s immediate stress reactions (i.e., the physiological fight-or-flight response), by altering the timecourse of the reaction or the magnitude [35,36,37,38]. Here, we found effects (although small) of the parasite on the cardiac stress reactions of passalus beetles in two experiments. In the first experiment, parasitized beetles tended to have an overall slower cardiac stress reaction (Figure 3), and in the second experiment, the magnitude of the stress reaction (i.e., HR elevation) was slightly lower than in non-parasitized beetles (34% vs. 45%; Table 3). Taken together, these results suggest the parasite causes a modest reduction in the ability of its host to react accordingly to stress stimuli. There are several possible explanations for why this might be happening. One that we favor is that this nematode acts as an energy drain to the host, so that limited energy is available during periods of high demand, such as during acute stress. Aside from reduced energy availability, another explanation for the parasite results is that it (the parasite) perhaps causes a state of chronic stress to its host, and we know chronic stress in vertebrate animals can reduce physiological reactions to acute stressors [39,40]. This results from the overstimulation of the stress reaction mechanism itself, to the point where it begins to attenuate.

Prior work in our lab using this host–parasite system also indicated how this nematode can negatively affect the immune activation that takes place during an acute stress reaction [22]. In that study, acute stress (mechanical tumbling) led to an increase in circulating hemocyte abundance in healthy beetles, but in parasitized beetles the hemocyte proliferation following stress was significantly reduced. Results from the current project help to explain this effect, since we now show that the stress-induced heartrate elevation in parasitized beetles is also reduced. Lower cardiac output would lead to less hemolymph distribution during stress, and by extension, fewer hemocytes being distributed during the stress event. Also of note from this prior work was that there was an attempt made to examine the effects of the nematode on ‘stressed’ beetle heartrates, and surprisingly, no effect was found [22]. However, in retrospect this probably had to do with the timing of the measurements; heartrates were measured before and after 10 min of mechanical stress. As the results here show, this parasite appears to exert most of its influence actually *during* the stress reaction, and less so immediately following the stressor.

What would be the consequences of parasitized beetles having a slower or reduced stress response? There are a variety of events in the lives of not just beetles, but all vertebrate and invertebrate species where there is a need for rapid, and effective stress reactions. These include fighting conspecifics to defend mates or territories, evading predators, and responding to immune challenges and/or wounds. The case of territorial defense might be of particular importance here; passalus beetles are known to defend their log cavities, and brood, from invading beetles [41,42,43], where they can engage in intense, and presumably energy-demanding fights. Prior work in our lab demonstrated that beetles harboring nematode parasites win slightly fewer fights than do healthy beetles [21], and their fights are less intense, which is consistent with the idea that the *C. passali* nematode limits energy availability during times of high demand, such as during a stressful event. Similarly, other work in our lab showed parasitized beetles have a small reduction in physical strength during a stress scenario [23].

It is important to consider that the parasite had no measureable impact on baseline heartrate of the beetles we tested in either experiment, which suggests that host metabolism and energy utilization are unaffected by it at the resting state. Heartrate is widely-considered a rough proxy for metabolism and energy use in humans and other animals e.g., [44,45]. Thus, this result is somewhat surprising given that the nematodes presumably consume nutrients from the hemolymph they live in (and which would otherwise go to the host). This implies that the parasite does not cause its host to ‘work harder’ to compensate for the nutrient losses by ramping up metabolism, at least in the resting state. In retrospect, this conclusion is consistent with other observations of this parasite. For example, there is no evidence that parasitized beetles suffer a cost in terms of growth, since adult body size is not lower in parasitized beetles [46]. The fact that this nematode is highly prevalent within collections of beetles [16,19] is another sign that it has little outward negative impact.

One admitted drawback to this study is our exclusive use of naturally-parasitized beetles collected in the wild. Even the beetles in our first experiment, which were reared in captivity, had initially been obtained as wild grubs, some of which were parasitized. Thus, we drew conclusions on the effects of this parasite based on comparisons of parasitized and non-parasitized beetle groups, but we are aware that other interpretations could be made. For example, beetles that tend to become parasitized may be inherently different (in terms of health) than those that are not parasitized. Ideally, the question of how this parasite (or any parasite) affect heartrates of its host would be best examined experimentally, using individuals inoculated with precise numbers of parasites, at the same developmental stage, etc. However, the many current unknowns about this parasite (its taxonomy, biology, mode of transmission, etc.) would make it difficult to conduct such an experiment until such information is obtained.

Efforts in our lab have been primarily focused on understanding the impact of this nematode on its host. Given our work thus far [21,23,28,46], combined with the current results, a picture is becoming clear regarding the true impact of this parasite. It seems the nematode, *C. passali* appears to most influence its beetle host during times of stress and intense energy-demanding activities, but otherwise has minimal effects on the day-to-day lives of the horned passalus, *O. disjunctus*. However, it is unclear at this time if the reduction in stress-related cardiac output caused by the parasite is the key to this energy drain (i.e., causing less hemolymph distribution to body compartments during stress), or if it is merely one of many bodily functions that are diminished because of low energy availability during stress. Continued investigations into this host–parasite system will hopefully provide answers to this and many other related questions about this intriguing parasite.

## 5. Conclusions

Results of these experiments lead us to conclude that the nematode parasite, *Chondronema passali*, conveys a subtle, but overall negative, impact to its host’s physiological reaction to acute stress. By tracking the host cardiac reaction before, during and after stress (restraint) in horned passalus beetles (*Odontotaenius disjunctus*), we found no effects of parasite infection on host baseline heartrates, but during the stressor those beetles with parasites showed a less-robust cardiac reaction than non-parasitized beetles; their heartrates did not increase as fast in one experiment and did not increase as high in the other experiment. Reducing the cardiac response during stress would result in reductions in hemolymph distribution to muscles or organs during the fight-or-flight reaction, and could result in costly delays in behavioral response times when the host is faced with imminent danger. Other work in our lab has shown similar results; that the greatest (physiological) impact of this parasite is during times of stress. This idea argues that researchers studying other parasites in insects (or vertebrates) should be cautious before concluding that a parasite does not impact its host; as seen here, the true cost of a parasite may only be observed when the host is under duress.

## Figures and Tables

**Table 1:** Magnitude of heartrate reactions prior to and during restraint stress in *O. disjunctus* from both experiments. In experiment 1, we used adults that had been reared in our lab from young larvae. In experiment 2 we used adults collected in the wild. *p* values denote significant differences between *experiments* based on Mann-Whitney U tests.

Heartrate Measurement	First Expt. (19 Reared)	Second Expt. (77 Wild)	Z	*p*
Initial HR (beats/min)	65.5 (1.7)	54.9 (10.2)	4.8	<0.0001
Max Stressed HR (beats/min)	81.5 (2.0)	74.2 (16.1)	3.3	0.001
Max Stressed HR (%)	24.5 (4.6)	35.5 (15.9)	−3.4	<0.001

**Table 2:** Summary of models that examined effects of nematode parasite infection on beetle heartrate before, during, and following stress application in the first experiment (*n* = 19 beetles).

**Baseline Heartrate**				
**Predictor**	**df**	**MS**	**F**	***p***
Mass	1	0.63	0.18	0.6815
Parasite	1	0.30	0.08	0.7776
Error	15	3.57		
**Stress Heartrate**				
**Predictor**	**df**	**MS**	**F**	***p***
Mass	1	25.45	3.97	0.0648
Parasite	1	145.00	22.64	0.0003
Error	15	6.41		
Time	4	4.16	1.78	0.1445
Time*Mass	4	5.14	2.20	0.0796
Time*Parasite	4	16.70	7.15	0.0001
Error	60	2.33		
**Recovery Heartrate**				
**Predictor**	**df**	**MS**	**F**	***p***
Mass	1	2.09	0.10	0.7541
Parasite	1	7.77	0.38	0.5476
Error	15	20.53		
Time	34	24.25	14.54	0.0000
Time*Mass	34	1.82	1.09	0.3335
Time*Parasite	34	2.31	1.38	0.0764
Error	510	1.67		

**Table 3:** Comparison of mean heartrate parameters between parasitized and non-parasitized beetles in the second experiment (*n* = 77 wild beetles).

Heartrate Measurement	Non-Parasitized Beetles (*n* = 14)	Parasitized Beetles (*n* = 63)
Baseline HR (beats/min)	51.1 (8.1)	55.7 (10.4)
Maximum Stressed HR (beats/min)	74.3 (16.6)	74.2 (16.1)
Maximum Stressed HR (%)	44.6 (17.1)	33.5 (15.0)

**Table 4:** Summary of ANOVA models examining effects of nematode parasite on baseline and maximum HR elevation (as a percentage of the initial HR) during stress application in experiment 2 (*n* = 77 beetles). Maximum stressed HR (beats/min) was not statistically evaluated for parasite effects (but see Table 3).

**Baseline Heartrate**				
**Predictor**	**df**	**MS**	**F**	***p***
Mass (g)	1	25.63	0.25	0.6185
Parasite	1	205.89	2.01	0.1605
Error	74	102.46		
**Stress Heartrate**				
**Predictor**	**df**	**MS**	**F**	***p***
Mass (g)	1	395.66	1.69	0.1975
Parasite	1	1620.75	6.93	0.0103
Error	74	233.94		

## Supplementary Materials

The following are available online at www.mdpi.com/2075-4450/8/4/110/s1, Video S1: Passalus beetle dorsal vessel.
